# Treatment Regret, Mental and Physical Health Indicators of Psychosocial Well-Being among Prostate Cancer Survivors

**DOI:** 10.3390/curroncol28050333

**Published:** 2021-10-02

**Authors:** Cassidy Bradley, Gabriela Ilie, Cody MacDonald, Lia Massoeurs, Vo Jasmine Dang Cam-Tu, Robert David Harold Rutledge

**Affiliations:** 1 Department of Community Health and Epidemiology, Dalhousie University, Halifax, NS B3H 1V7, Canada; cs724384@dal.ca (C.B.); CodyMacDonald@dal.ca (C.M.); Lia.Massoeurs@dal.ca (L.M.); 2 Department of Urology, Dalhousie University, Halifax, NS B3H 4R2, Canada; Rob.Rutledge@nshealth.ca; 3 Department of Psychology and Neuroscience, Dalhousie University, Halifax, NS B3H 4R2, Canada; 4 Department of Radiation Oncology, Dalhousie University, Halifax, NS B3H 4R2, Canada; Jasmine.Vo@dal.ca; 5 Faculty of Medicine, Dalhousie University, Halifax, NS B3H 1V7, Canada

**Keywords:** prostate cancer, treatment regret, quality of life, cancer survivorship, emotional well-being, functional well-being, spiritual well-being, social well-being, patient autonomy

## Abstract

Prostate cancer (PCa) patients and survivors are at high risk of mental health illness. Here, we examined the contribution of treatment regret, mental and physical health indicators to the social/family, emotional, functional and spiritual well-being of PCa survivors. The study assessed 367 men with a history of PCa residing in the Maritimes Canada who were surveyed between 2017 and 2021. The outcomes were social/family, emotional, functional and spiritual well-being (FACT-P,FACIT-Sp). Predictor variables included urinary, bowel and sexual function (UCLA-PCI), physical and mental health (SF-12), and treatment regret. Logistic regression analyses were controlled for age, income, and survivorship time. Poor social/family, emotional, functional and spiritual well-being was identified among 54.4%, 26.5%, 49.9% and 63.8% of the men in the sample. Men who reported treatment regret had 3.62, 5.58, or 4.63 higher odds of poor social/family, emotional, and functional well-being, respectively. Men with low household income had 3.77 times higher odds for poor social/well-being. Good mental health was a protective factor for poor social/family, emotional, functional, or spiritual well-being. Better physical and sexual health were protective factors for poor functional well-being. Seeking to promote PCa patients’ autonomy in treatment decisions and recognizing this process’ vulnerability in health care contexts is warranted.

## 1. Introduction

While the prostate cancer (PCa) mortality rate has been decreasing in high income countries over the past 20 years, the incidence and burden of this disease are increasing globally [1]. In fact, between 1990 to 2015, the disability adjusted life-years attributable to PCa increased by 90% [2]. With increased survivorship and burden of disease, it is important to consider the quality of life (QoL) of PCa patients during the survivorship cancer journey. There is limited research investigating how treatment regret, uro-oncological function (urinary, bowel, and sexual), and physical and mental health may affect the social/family, emotional, functional and spiritual well-being of this population following treatment. This is especially important given recent evidence showing over 1.5 times higher odds for mental health illness compared to men affected by other cancers or over 2 times higher odds compared to men who never had a cancer diagnosis in their lifetime [3,4,5]. Steginga et al. (2004) found that as many as 60% of men with PCa experience psychological distress after treatment [6]. Previous studies found that rates of depressive symptoms among PCa survivors range from 16% to as high as 30% [7,8,9,10,11,12], whereas for the general population of men of a comparable age group it is less than 9% [13]. Moreover, Watts et al. (2014) found that anxiety prevalence rates among men with PCa range from 15% to 27%, while for men of a comparable age range in the general population they are less than 6% [13]. While these findings are concerning, they are, however, not surprising.

While PCa-specific survival is high, at up to 98.8% survival at 10 years after initial diagnosis [14], the side effects that accompany the various treatment modalities used in modern medicine and treatment decision making, that affect QoL, are significant [15]. For this reason, it has become increasingly important to consider QoL outcomes associated short and long term. Short term QoL outcomes have been extensively documented, but only a few reports have evaluated the long term (beyond 10 years) QoL and survival associated with different treatment options [16]. QoL is a challenging concept to define, yet the principle of measuring the impact of an illness and active or non-active treatments on the patient’s well-being, rather than simply focusing on health status or survival, is now well accepted in healthcare research. In this paper we are concerned with predetermined set of QoL domains or health related constructs of well-being such as emotional, functional, social/family and spiritual well-being assessed through validated questionnaires where patients and survivors are presented with each domain and are asked to rate their performance in each area [17]. Other QoL indicators, not assessed here, include an assessment where each patient nominates significant QoL domains according to their individual preferences and rate their level of satisfaction with each domain [18]. In prostate cancer, the most significant functional and physical health related side effects that affect the QoL of patients are sexual, urinary, and bowel problems [11,19,20], fatigue [21], problems sleeping [9] and cardiovascular events [22]. Psychosocial side effects following treatment for PCa have also been identified and include mental health illness [3,5,10,23,24,25], poor attendance to support groups [9], low income [4], relationship difficulties [9,10] and being single, separated or divorced [26].

While the evidence for the impact of treatment regret on the psychosocial well-being aspects of QoL among prostate cancer survivors is scarce, its contribution may be important. Diefenbach and Mohamed (2009) found that PCa survivors who were regretful about their treatment choice(s) had lower QoL compared to those who were not regretful [27]. Previous studies have also found that men who had a passive role in treatment decision-making had higher treatment regret than men who assumed an active or collaborative role [28], and that among those who participated in medical decision-making, 94% did not experience treatment regret. Further, Davison and Goldenberg (2003) found that emotional functioning was significantly better at follow-up among PCa patients who participated in their treatment decision than those who did not [29]. This suggests that treatment regret may play a critical role in emotional well-being, which is an important determinant of QoL.

A review by Colloca and Colloca (2015) found that in the early stages of PCa survivorship, psychological and physical needs are paramount, while in the later stages, physical symptoms, social and spiritual needs predominate among survivorship needs [30]. A review of the literature has shown that spiritual well-being, is just as an important psychosocial marker of QoL among PCa survivors as social, emotional, functional and physical wellbeing, although more research is needed to understand its psychological and health determinants among prostate cancer survivors [31,32]. Understanding prostate cancer survivorship needs and the psychosocial and health determinants of QoL among this population is crucial, as it may serve to provide more individualized, multidisciplinary, patient-centered survivorship care. Further, this understanding is paramount for informing the development of patient education and empowerment programs [33].

In this study we examine the contribution of treatment regret along with physical and psychological indicators to the social/family, emotional, functional and spiritual well-being of PCa survivors, while controlling for age, household income and survivorship time. To our awareness, this is the first study to assess the combined contribution of these predictors of quality-of-life indicators.

## 2. Methods

This analytical sample from this cross-sectional study was composed of 367 PCa survivors (mean age = 68.50 years, SD = 7.18, range 47 to 88 years old) who took an online survey assessing their QoL between May 2017 and December 2021. Males of age that spoke English, resided at the time they took the survey in either New Brunswick, PEI or Nova Scotia, had a history of localized prostate cancer diagnosis, and had an email address were eligible to participate in this study. Participants were recruited through printed materials that were advertised in the Urology and Radiation Oncology Clinical offices throughout the Maritimes, as well as Prostate Cancer Support groups in the region. This was a convenience sample. Interested participants were asked to either contact our research coordinator, and review the study, or access the on-line informed consent link directly. Once they accessed the on-line link, they first asked to complete an e-informed consent on their own or review the study with our research coordinator. Once participant e-consent was obtained which included providing their email address and health card number for the study, participants were taken to the on-line survey questions. This was a random convenience sample. Among the patients who contacted our office, 68% responded. The survey duration was approximately 25 min. Survey procedures were in accordance with the ethical standards of the responsible committee on human experimentation and with the Helsinki Declaration of 1975, as revised in 2000. The survey data was stored in the REDCap (Research Electronic Data Capture) online database supported by the Nova Scotia Health Authority and according to the Patient Health Information Act (PHIA). Approval for this study was granted by the Nova Scotia Health Authority (NSHA) Horizon New Brunswick, and Prince Edward Island Research Ethics Boards (project # 1021455). Data that was generated by REDCap and resided on the private and secure NSHA servers, and it was deidentified (for the anonymity and confidentiality protection of the participants) prior to being accessed by to our team for statistical analysis.

### 2.1. Measures

#### 2.1.1. Outcomes

Social/family, emotional and functional well-being were assessed using the Functional Assessment of Cancer Therapy—Prostate (FACT-P). This is a 39-item 5-point Likert-type scale validated to measure health related quality of life (HRQoL) in PCa patients aged 18 years and older, over the past 7 days [34]. The social/family emotional and functional well-being subscales consist of 7, 6 and 7 items, respectively. Questions in the social well-being subscale assesses concepts such as social support from friends and family, sense of closeness to friends, emotional support from family, family acceptance of illness, satisfaction with family communication about illness, closeness to one’s partner and satisfaction with sex life. The emotional well-being subscale assesses participants’ sense of sadness, satisfaction with how they are coping with illness, sense of hope in fighting their illness, nervousness, worry about dying and worry about the condition worsening. The functional well-being subscale assesses participants’ abilities to work, sense of fulfilment from work, ability to enjoy life, acceptance of illness, sleep quality, enjoyment of activities done for fun and contentment with QoL. Scores on each of these subscales are obtained by summing the scores from each question of the subscale, with reverse coding for negatively worded questions. Responses on each of the three subscales ranged from 0 (“Not at all”), 1 (“A little bit”), 2 (“Somewhat”), 3 (“Quite a bit”) to 4 (“Very much”). A binary variable based on mean scores for each subscale was created to indicate good well-being if the mean score for each subscale was 3 or below (coded 0), or poor well-being if the mean score for each subscale was above 3 (coded 1). This is in keeping with evidence from Jeong and Lee (2016) that 5-point Likert scales dichotomized into 1–3 and 4–5 performed well compared to their original 5-point scale (similar *p* values for significance with each predictor when correlated using both dichotomous and continuous coding) [35]. The Pearson Correlation coefficients between the binary variable created and the continuous variable for each of the subscales are as follows: Social/Family (−0.743), Emotional (−0.805) and Functional (−0.799). This indicates good overall correlations between the binary variables (which were reversely coded such that a 0 indicates good well-being and a 1 indicates poor well-being) and continuous variables (for which a higher score indicates increased well-being). Cronbach’s alpha for social/family, emotional and functional in the data reported here were 0.841, 0.786 and 0.882, respectively, which indicate overall good internal reliability and are comparable to those reported in the literature [34,36,37]. Concurrent validity for the FACT-P has been confirmed by the ability to discriminate patients by disease stage, performance status, and baseline prostate-specific antigen (PSA) level [34]. Moreover, the FACT-P has been validated in several languages, making it widely accessible [38].

Spiritual well-being was measured using the Functional Assessment of Chronic Illness Therapy-Spiritual Well-Being (FACIT-Sp-12) which is a 12-item 5-point Likert-type scale designed for patients with a chronic illness, aged 18 years and older [39]. Questions assessed participants on 3 subscales of spirituality: meaning, faith and peace over the past 7 days. Responses on each of the three subscales ranged from 0 (“Not at all”), 1 (“A little bit”), 2 (“Somewhat”), 3 (“Quite a bit”) to 4 (“Very much”). An overall score for spiritual well-being is obtained by summing the mean scores from each subscale. Participants were classed as “good” if their overall score is 3 or below, and “poor” if their mean score is above 3. As with the FACT-P, this categorization is in keeping with evidence from Jeong and Lee (2016) that 5-point Likert scales dichotomized into 1–3 and 4–5 performed well compared to their original 5-point scale [35]. The Pearson correlation coefficient correlating the binary and continuous variables form Spiritual Well-Being is −0.777. This indicates a good correlation between the binary variable (which was reversely coded such that a 0 indicates good well-being and a 1 indicates poor well-being) and continuous variables (for which a higher score indicates higher well-being). The Cronbach’s alpha for the items in the current data set was 0.882, indicating good internal reliability and it is comparable with coefficients reported in the literature [40,41]. FACIT-Sp-12 is established as a reliable and valid measurement of spiritual well-being that may be particularly useful in assessing the role of both religious and non-religious spiritual well-being in health-related QoL [39,40,41].

#### 2.1.2. Predictors

Sexual, bowel and urinary functioning were assessed using the UCLA Prostate Cancer Index (function) which is a 17-item scale used to measure HRQoL among men with localized PCa [42]. The recall period assessing each domain was 4 weeks. The sexual function scale had 8 questions and assessed levels of sexual desire, ability to have an erection, ability to reach orgasm, quality of erections, frequency of erections, frequency of awakening with an erection, vaginal or anal intercourse history, and overall sexual function. The bowel function subset was comprised of 4 questions and asked participants about frequency of rectal urgency, frequency of loose stool, bowel movement distress and frequency of crampy pain in the abdomen or pelvis. The urinary function subset was composed of 5 questions and assessed frequency of urine leakage, degree of urinary control, number of pads or adult diapers used per day, degree their problem with dripping urine or wetting their pants and urine leakage interfering with sexual activity. Items for urinary, bowel and sexual function had responses with associated numeric scores of either 1–4, 1–5 or 1–6. These scores were then recoded to an associated value between 0–100 provided by the authors in the scoring instructions, and mean scores for each domain were calculated [42]. The Cronbach’s alpha for sexual, bowel, and urinary function in this sample was 0.875, 0.582 and 0.777, all of which indicate good internal reliability. The UCLA-PCI is a reliable and valid measure in males with and without PCa, with test–retest reliability ranging from 0.66 to 0.93, and internal consistency ranging from 0.65 to 0.93 [43].

Physical- and mental-health-related QoL was assessed using the SF-12v2 which consists of 12 questions that assess physical and mental health, intended for adults of the general population [44,45]. The 12 items assess what respondents are able to do, how they feel, and how they evaluate their health over the past 4 weeks. These questions are used to assess eight health concepts: physical functioning, role limitations due to physical health problems, bodily pain, general health, vitality, social functioning, role limitations due to emotional problems and mental health. The instrument has been validated across a number of chronic diseases and conditions. The response options for each item are specific to the question, and include responses such as description of health, degree of limitation of specific symptoms, yes/no options, degree of interference and amount of time spent with specific symptoms. In order to obtain overall Physical Component Summary scores (PCS) and Mental Component Summary Scores (MCS), indicator variables for item response choices must be created (scored 1 for selected and 0 for not selected), for a total of 35 indicator variables. Items #1, #8, #9 and #10, must be reverse coded and indicator variables must be created for each item response. Finally, MCS and PCS scores are computed by multiplying each indicator variable by its respective mental/physical regression weight provided by the authors in the scoring criteria guideline [45] and summing the 35 products. Scores range from 0–100, with higher scores indicating better health. The Cronbach’s alpha for the PCS and MCS in this sample was found to be 0.86 and 0.89, respectively, indicating high internal reliability, which is comparable with the coefficients previously reported in the literature [46,47]. Both the PCS and MCS correlate highly with those of the long version of the measure, SF-36v2 (multiple R squares of 0.911 and 0.918) [44].

Treatment regret: In order to assess for the presence or absence of treatment regrets, participants were asked “Do you have any regrets with regards to the treatment you received for your prostate cancer diagnosis?”. Responses were coded as “No” = 1 and “Yes” = 2.

*Demographic variables:* Demographic variables included as covariates in the study were age, household income in the past 12 months (coded 1 for less than CAD 50,000, 2 for CAD 50,000 to 100,000, and 3 for over CAD 100,000) and time elapsed between diagnosis (the date the patient was told they had prostate cancer) and survey (date of the survey) measured in months, which are covariates shown previously in the literature to be significant associates of QoL among prostate cancer survivors [3,4,5,10].

### 2.2. Statistical Analyses

Statistical analyses were performed using SPSS V26. Before beginning the analyses, the assumptions of logistic regression were checked and found tenable. Cross tabulations were used to assess the association between each of the four binary outcomes (social, emotional, functional and spiritual well-being) and each of the 6 predictors (urinary, bowel, and sexual function, physical and mental health function, treatment regret) as well as each individual covariate (age, household income and time elapsed between diagnosis and survey). Multivariate logistic regression analyses were used to model each of the well-being domains (social/family, emotional, functional and spiritual) based on the six prediction and the covariates. Social/family and emotional well-being outcomes had 1.4% missing data, the functional well-being outcome had 1.6% missing and the spiritual well-being outcome had 2.2% missing. After listwise deletion the analytical sample for the multivariate logistic regressions was 362 for social/family and emotional well-being, 361 for functional well-being and 359 for spiritual well-being.

## 3. Results

Table 1 presents the associations between the six individual predictors and the three covariates and the outcome variables. A total of 54.4% of men from the sample screened positive for poor social/family well-being. Poor social/family well-being was positively associated with a household income below CAD 50,000 (OR = 3.77, 95% CI: 1.12–12.65) and the presence of treatment regret (OR = 3.88, 95% CI: 1.58–9.53). The presence of good mental and physical health, good bowel and sexual function were each negatively related to poor social/family well-being (OR = 0.88, 95% CI: 0.84–0.93; OR = 0.91, 95% CI: 0.87–0.95; OR = 0.98, 95% CI: 0.96–0.99; OR = 0.98, 95% CI: 0.98–0.99, respectively). Poor emotional well-being was present among 26.5% of the men in the sample and was positively associated with treatment regret (OR = 5.42, 95% CI: 2.41–12.19). The presence of age, good mental and physical health, good bowel and sexual function were each negatively related to poor emotional well-being (OR = 0.96, 95% CI: 0.92–0.99; OR = 0.87, 95% CI: 0.83–0.91; OR = 0.94, 95% CI: 0.90–0.98; OR = 0.98, 95% CI: 0.97–0.99; OR = 0.98, 95% CI: 0.95–0.99, respectively). Poor functional well-being was present among 49.9% among respondents and was positively associated with treatment regret (OR = 4.18, 95% CI: 1.76–9.93). The presence of good mental and physical health, good urinary, bowel and sexual function were each negatively related to poor functional well-being (OR = 0.84, 95% CI: 0.79–0.88; OR = 0.88, 95% CI: 0.84–0.92; OR = 0.99, 95% CI: 0.97–0.99; OR = 0.97, 95% CI: 0.96–0.98; OR = 0.98 95% CI: 0.96–0.99, respectively). Poor spiritual well-being emerged for 63.8% of respondents and was positively associated with treatment regret (OR = 3.02, 95% CI: 1.17–7.79). The presence of good mental and physical health, age and survivorship time were each negatively related to poor spiritual well-being (OR = 0.85, 95% CI: 0.80–0.90; OR = 0.94, 95% CI: 0.90–0.98; OR = 0.95, 95% CI: 0.91–0.98; OR = 0.99, 95% CI: 0.98–0.99, respectively).

The multivariate logistic regression model predicting the presence of poor social/family well-being based on the six predictors (urinary, bowel and sexual function, treatment regret, physical and mental health) and three covariates (age, household income and survivorship time) was statistically significant X^2^(10) = 35.77, *p* < 0.001, with a Nagelkerke’s R^2^ = 0.32, and Hosmer and Lemeshow test showing model stability (X^2^(8) = 5.52, *p* > 0.05) (See Table 2). This model was accurate for 69.5% of participants. The odds for poor social/family well-being were 3.77 (95% CI: 1.12–12.65) times higher among men with lowest past year household income, and 3.62 (95% CI: 1.16, 11.36) times higher among those who reported treatment regret. Good mental health was a protective factor for social/family well-being (OR = 0.90, 95% CI: 0.82–0.99).

The multivariate logistic regression model predicting the presence of poor emotional well-being based on the contribution of all six predictors and the three covariates was also statistically significant X^2^(10) = 42.95, *p* < 0.001, with a Nagelkerke’s R^2^ = 0.42, and Hosmer and Lemeshow test showing model stability (X^2^(8) = 7.23, *p* > 0.05) (See Table 2). This model was accurate for 84.0% of participants. The odds for poor emotional well-being were 5.58 (95% CI: 1.75, 17.75) times higher among men who reported having treatment regret. Good mental health was a protective factor against poor emotional well-being (OR: 0.80, 95% CI: 0.71–0.90).

The multivariate logistic regression model assessing the presence of poor functional well-being based on the six predictors and three covariates was statistically significant X^2^(10) = 63.23, *p* < 0.001, with a Nagelkerke’s R^2^ = 0.51, and Hosmer and Lemeshow test showing model stability (X^2^(8) = 12.36, *p* > 0.05) (See Table 2). This model was accurate for 56.4% of participants. The odds for poor functional well-being were 4.63 (95% CI: 1.40–15.36) times higher among men who reported having treatment regret. Good sexual function (OR = 0.98, 95% CI: 0.96–0.99), having a longer time elapsed between diagnosis and survey completion (OR: 0.99, 95% CI: 0.98–0.99), the presence of good mental (OR: 0.77, 95% CI: 0.67–0.87) and physical health (OR: 0.82, 95% CI: 0.72–0.92) were protective factors for poor functional well-being.

Lastly, the multivariate logistic regression model predicting the presence of poor spiritual well-being based on the contribution of the six predictors and three covariates was statistically significant X^2^(10) = 35.39, *p* < 0.001, with a Nagelkerke’s R^2^ = 0.32, and Hosmer and Lemeshow test showing model stability (X^2^(8) = 6.63, *p* > 0.05) (See Table 2). This model was accurate for 71.8% of participants. Protective factors against poor spiritual well-being were low or moderate household income in the previous year (OR: 0.18, 95% CI: 0.48, 0.67 or OR: 0.30, 95% CI: 0.10–0.88, respectively) longer time elapsed since diagnosis (OR: 0.99, 95% CI: 0.98–0.99), and good mental (OR: 0.83, 95% CI: 0.73–0.93) and physical health (OR: 0.85, 95% CI: 0.76–0.94).

## 4. Discussion

Here we investigated the contribution of treatment regret along with physical and psychological indicators to the social/family, emotional, functional and spiritual well-being of PCa survivors. When all variables in the model were held constant, the odds of poor social/family, emotional and functional well-being among men who regretted their form of treatment for their PCa diagnosis were 3.77, 5.58, 4.63 times greater compared to men who reported no regret. To our knowledge, this is the first study to assess the contribution of treatment regret to these specific aspects of QoL. Treatment regret has been previously associated with poor urinary and sexual function following treatment, particularly following surgery, at 6 to 12 months post-treatment [27]. In 2015 a systematic review of the literature identified 28 studies examining treatment regret and found that the most common reasons for treatment regret were the presence of poor urinary and sexual function, the choice of surgery over other active forms of treatment, and toxicity side effects associated with radiation therapy [48]. Here we extend the literature and show that treatment regret is associated with poor social/family, emotional and functional well-being of survivors. These findings highlight the importance of better informing men about treatment options and maximizing patient and family understanding of the PCa treatment options available, which may help mitigate long-term treatment regret [48,49]. Decision making aids, which provide accurate as well as easy-to-understand information for patients to help navigate the decision-making process have been shown to be effective and are the most commonly used ways for minimizing treatment regret [50].

Patient-centred care, including patient autonomy must also be prioritized among PCa survivors as communication that values patient autonomy is strongly associated with satisfaction with care [51]. As emphasized by Shervach et al. (2019) clinicians should discuss not only the efficacy of the various PCa treatment options, but also how they may impact QoL and allow patients to weight in the decision-making process as well as make voluntary choices about potentially life-changing side effects, based on what aspects of QoL they value [52]. Moreover, Sanda et al. (2018) recommend that shared decision-making between clinician and patient be utilized with consideration of cancer severity, patient values and preferences, life expectancy, pre-treatment general and genitourinary function, expected post-treatment function and potential for salvage treatment in the decision [53]. This patient-centred approach undoubtedly discourages some inappropriate top-down (doctor making decisions for the patient) treatment decisions and protects some patients from unwanted interventions, by allowing men to, for example, decline surgery that they may consider more burdensome than beneficial. Enabling patients to be autonomous in the treatment modality decision making process, and make informed decisions emphasizes the importance of the individuals’ understandings of health care interventions and supports the development and use of potentially autonomy-enhancing patient decision aids [54].

Low social economic status measured through past year household income was a statistically significant predictor of poor social/family well-being among PCa survivors when physical and mental health, urinary, bowel and sexual function as well as age and survivorship were held constant. This is the first study to connect low socio-economic status and poor social/family well-being among PCa survivors. Evidence from Short and Mallonee (2006) suggests that among cancer patients, those with high-income have better prognoses, and live with better QoL throughout survivorship than those with low income [55]. Results also show that higher socioeconomic status defined as having reported a household income above CAD 100,000 was associated with poor spiritual well-being. This is consistent with previous research which has reported that low income usually is associated with better spiritual well-being and higher religiosity [56,57]. Another study, by Zavala et al. (2009) examined how spirituality is associated with HRQoL among low-income men with metastatic PCa and found that greater spirituality was associated with better HRQoL and psychosocial function [58].

Previous findings have identified low socio-economic status as a significant socio determinant of risk, incidence, poor survival, as well as poor mental health among PCa patients [4,59]. Good mental health was a protective factor against social/family, emotional, functional as well as spiritual well-being. This is the first time the associate contribution of mental health was assessed in relation to these QoL indicators among PCa survivors. These results are not surprising given recent population-based data which has uncovered that mental distress is a silent epidemic [3,4,5,7,25,33], and critical marker of QoL among PCa survivors [60]. Physical health was also a statistically significant predictor in the models assessed for functional and spiritual well-being. This is not surprising in light of previous studies indicating that spiritual well-being correlates with other QoL including physical well-being and psychosocial health, among early-stage PCa patients [31,61,62,63]. These results extend results from Holt-Lunstad (2011) who found that spiritual well-being was associated with better cardiovascular health [64]. Physical health and functional well-being have long been shown to affect one another [65]. For these reasons, patients should be encouraged to participate in activities that target physical function throughout PCa survivorship, which may include pelvic floor physiotherapy and/or patient empowerment programs [33]. This study, however, shows how mental health was a contributor to all aspects of QoL measured here, but not the same was observed for physical health. Although our understanding of the mental and physical health benefits for PCa survivors is increasing, more research, as the study we have undertaken here, is needed to parse out their contribution to the various aspects of QoL. One implication of the results we report here is that it points to the importance of examining healthcare providers’ knowledge of the physical health guidelines for cancer patients and their recommendations for use with PCa survivors.

Urinary function did not emerge as a statistically significant predictor of social/family, emotional, functional and spiritual well-being in the predictive models we assessed here, although it was, when individually assessed, statistically significantly associated with functional well-being. Bowel function was, on its own, associated with social/family, emotional and functional well-being but its contribution to the predictive models when the other predictors and covariates were held constant, also disappeared. Results here indicate that psychosocial factors such as mental health and treatment regret seem to weight in more heavily in predicting these aspects of QoL. Sexual function was associated with social/family, emotional and functional well-being when assessed alone, but only contributed to functional well-being when the other predictors and covariates were entered in the model. The contribution of sexual function to this aspect of QoL has been previously documented [66]. This is in keeping with results from Lewis et al. (2004), who found a strong association between self-rated health, and sexual well-being [67]. Decreased sexual function is often very distressing to men [68], which may in turn lead to lower self-perceived functional well-being. Our results may suggest that treatment efforts aimed at improving sexual function are a health priority throughout PCa survivorship, which further highlights the importance of programs such as pelvic floor physiotherapy and patient sexual education related to their treatment side effects [33]. Urinary, bowel and sexual function when assessed on their own or with the other predictors and covariates were not statistically significantly associated with spiritual well-being. Physical and mental health, longer survivorship time, and lower household income were all protective factors for poor spiritual well-being.

Longer survivorship time emerged as a statistically significant predictor in assessing both functional and spiritual well-being. Indeed, the longer the time elapsed between diagnosis and the time when the survey was administered the lower the probability of poor functional and spiritual well-being among our survivors. It is possible that as survivors get more adjusted to their new life circumstances, and side effects, post-treatment, as they recover from some of the side effects, that their perception of their functional well-being and their connection to their spiritual believes also improves. More research is needed to understand how these various predictors affect the various aspects of QoL we examined here in the various stages of survivorship during the PCa journey.

Limitations of this study include that it is retrospective and thus is subject to recall bias, as well as volunteer bias due to the voluntary nature of participation. Moreover, the data is self-reported and may be subject to social-desirability bias. Survival bias may limit generalizability of results, as those patients with severe disease are less likely to enter the study, and those with metastatic disease were not eligible. Results indicate associations thus causality cannot be inferred. No baseline pre-treatment data is available. It would be interesting and relevant to study how the predictors we assessed affect the outcomes of the study over time as patients progress through treatment and the various stages of the survivorship period. Residual confounding may play a role in the analyses. For example, rural versus urban settings, or province where the treatment was given may play a role, although treatment is similar across all Maritimes provinces. Studies of larger sample size should consider controlling for these possible residual influences. While income is a traditional indicator of socioeconomic status, compositional approaches to socioeconomic status that include education and occupation are preferred [69]. Others have argued that these three indicators still fail to account for household contributions such as inheritances, savings, which can greatly improve an individual’s social and economic situation, or material depravation. Indeed, income alone may not be the most accurate proxy for overall socioeconomic status. A household income of less than CAD 50,000 would mean more to someone living alone in rural Maritimes than a couple living in urban central settings (e.g., Halifax downtown). Future studies may want to consider composite measures of socioeconomic status measures to better understand disparities in quality of life during prostate cancer survivorship [70]. While other researchers have used similar renditions of the question we used, to assess the presence of treatment regret, in past research [71,72], it could be argued that the question may have been leading. Future studies should consider the construction of validated questionnaire for the assessment of treatment regret and reassess these associations. The assessment of the reasons behind treatment regret when present, also merit further investigation. Lastly, although guided by the existing literature, the study we present here could be said to be exploratory in nature. Future studies with treatment regret as primary exposure variable should further explore the relationships we report here.

Despite these limitations, the results of this study provide an important contribution to our understanding of the role of physical and psychological indicators to social/family, emotional, functional and spiritual well-being. We note several strengths to this study. These include the utilization of different domains of QoL through the use of the FACT-P measure, independently used as outcomes, such as to allow us for a better understanding of the specific role of different physical, psychological and mental health indicators on QoL. The use of FACT-P adds strength to the study given that this QoL measure has been well validated and has demonstrated good internal consistency and reliability in evaluating QoL in patients with prostate cancer undergoing various forms of treatment [34,73,74]. The calculation of Cronbach’s alpha coefficients to ensure adequate internal reliability for each measure is another strength of the current study as well as the inclusion of treatment regret as a novel predictor of QoL. Dijkers (2003) proposed that QoL measures be viewed on a continuum dependent on the degree to which patients are allowed to individualize their responses based on their individual preference and value system [75]. The investigation we present here is based on one end of the continuum Dijkers (2003) defines, where our predictors speak to a set of predetermined QoL domains (functional, emotional, social/family and spiritual well-being) [75]. Each approach to QoL has its advantages and disadvantages, and together they could provide a more comprehensive data source that would be considerably more informative than either approach in isolation. Future studies should consider the addition of more individualized QoL measurement approaches.

Nonetheless, based on the results we report here the data suggests the need for multidisciplinary management (including mental health assessments during survivorship) and patient-centred care throughout PCa survivorship. The findings also point to the importance of informed decision making and the need for physician-patient discussions prior to deciding a course of action for prostate cancer treatment. The data we present here points to the importance of making sure patients are informed and taking part on their treatment decisions, as this process is not void of the patient’s quality of life during their cancer journey and may mitigate their long-term treatment regret.

## Figures and Tables

**Table 1:** Cross-tabulations assessing the relationship between social/family, emotional, functional and spiritual well-being outcomes and uro-oncological function, physical and mental health indicators, and demographic variables in a sample of men with a history of prostate cancer diagnosis from the baseline cycle of a Quality-of-Life Maritimes Survey administered, 2017–2020, *n* = 367.

	Good Social/Family Well-Being (*n* = 165) OR (95% CI)	Poor Social/Family Well-Being (*n* = 197) OR (95% CI)	Wald X^2^
Urinary function ^1^ (severity of lower urinary tract symptoms), UCLA ^2^, Mean (SD)	76.78 (20.77) 1.0 Reference	77.47 (21.61) 1.00 (0.992, 1.011)	X^2^(1) = 0.095
Bowel function ^1^ (severity) of bowel symptoms, UCLA ^2^, Mean (SD)	87.24 (15.5) 1.0 Reference	80.49 (18.64) 0.98 (0.96, 0.99) **	X^2^(1) = 11.55 **
Sexual function ^1^ (severity of disfunction), UCLA ^2^, Mean (SD)	32.77 (29.41) 1.0 Reference	21.05 (23.61) 0.98 (0.98, 0.99) ***	X^2^(1) = 12.88 ***
Physical health ^1^, SF-12 ^3^ Mean (SD)	49.32 (4.56) 1.0 Reference	46.73 (5.63) 0.91 (0.87, 0.95) ***	X^2^(1) = 19.68 ***
Mental health ^1^, SF-12 ^3^, Mean	47.24 (4.24) 1.0 Reference	44.26 (5.78) 0.88 (0.84, 0.93) ***	X^2^(1) = 24.62
Treatment regret			X^2^(1) = 8.73 **
Presence	8.4% 1.0 Reference	26.3% 3.88 (1.58, 9.53) **	
Absence	91.6% 1.0 Reference	73.7% 1.0 Reference	
Age, Mean, SD	68.97 (6.84) 1.0 Reference	68.19 (7.35) 0.984 (0.95, 1.01)	X^2^(1) = 0.975
Survivorship time (months) from diagnosis, Mean (SD)	67.82 (57.38) 1.0 Reference	59.19 (57.89) 1.86 (0.994, 1.00)	X^2^(1) = 1.75
Household Income			X^2^(2) = 11.70 **
*<CAD 50,000*	15.7% 1.0 Reference	33.1% 2.97 (1.54, 5.74) **	
*CAD 50,000–100,000*	48.5% 1.0 Reference	41.4% 1.20 (0.70, 2.06)	
*>CAD 100,000*	35.8% 1.0 Reference	25.5% 1.0 Reference	
	Good emotional well-being (*n* = 266) 1.0 Reference	Poor emotional well-being (*n* = 96) OR (95% CI)	Wald X^2^
Urinary function ^1^(severity of lower urinary tract symptoms), UCLA ^2^, Mean (SD)	78.04 (20.53) 1.0 Reference	74.74 (22.88) 0.99 (0.98, 1.00)	X^2^(1) = 1.70
Bowel function ^1^ (severity) of bowel symptoms, UCLA ^2^, Mean (SD)	84.98 (16.34) 1.0 Reference	79.45 (20.25) 0.98 (0.97, 0.99)	X^2^(1) = 6.25 *
Sexual function ^1^ (severity of disfunction), UCLA ^2^, Mean (SD)	29.04 (28.39) 1.0 Reference	18.76 (20.89) 0.98 (0.95, 0.99) **	X^2^(1) = 7.69 **
Physical health ^1^, SF-12 ^3^, Mean (SD)	48.36 (5.04) 1.0 Reference	46.66 (5.90) 0.94 0.90, 0.98) **	X^2^(1) = 6.88 **
Mental health ^1^, SF-12 ^3^, Mean	46.67 (4.79) 1.0 Reference	42.67 (5.70) 0.87 (0.83, 0.91) ***	X^2^(1) = 32.19 ***
Treatment regret			X^2^(1) = 16.68 ***
Presence	10.6% 1.0 Reference	39.1% 5.42 (2.41, 12.19) ***	
Absence	89.4% 1.0 Reference	60.9% 1.0 Reference	
Age, Mean, SD	69.10 (6.85) 1.0 Reference	66.98 (7.670 0.96 0.92, 0.99) *	X^2^(1) = 5.34 *
Survivorship time (months) from diagnosis, Mean (SD)	67.32 (58.08) 1.0 Reference	51.16 (55.27) 1.00 (0.99, 1.00)	X^2^(1) = 4.61
Household Income			X^2^(2) = 2.52
*<CAD 50,000*	22.8% 1.0 Reference	31.9% 1.47 (0.73, 2.94)	
*CAD 50,000–100,000*	46.6% 1.0 Reference	38.9% 0.876 (0.46, 1.67)	
*>CAD 100,000*	30.6% 1.0 Reference	29.2% 1.0 Reference	
	Good functional well-being (*n* = 181) 1.0 Reference	Poor functional well-being (*n* = 180) OR (95% CI)	Wald X^2^
Urinary function ^1^ (severity of lower urinary tract symptoms), UCLA ^2^, Mean (SD)	79.66 (19.81) 1.0 Reference	74.67 (22.35) 0.99 (0.97, 0.99) *	X^2^(1) = 4.84 *
Bowel function ^1^ (severity) of bowel symptoms, UCLA ^2^, Mean (SD)	87.93 (14.29) 1.0 Reference	79.33 (19.39) 0.97 (0.96, 0.98) ***	X^2^(1) = 18.27 ***
Sexual function ^1^ (severity of disfunction), UCLA ^2^, Mean (SD)	33.86 (29.87) 1.0 Reference	18.68 (21.16) 0.98 (0.96, 0.99) ***	X^2^(1) = 21.16 ***
Physical health ^1^, SF-12 ^3^, Mean (SD)	49.49 (4.15) 1.0 Reference	46.35 (5.88) 0.88 (0.84, 0.92) ***	X^2^(1) = 28.28 ***
Mental health ^1^, SF-12 ^3^, %	47.60 (3.94) 1.0 Reference	43.67 (5.80) 0.84 (0.79, 0.88) ***	X^2^(1) = 39.83 ***
Treatment regret			X^2^(1) = 10.50 **
Presence	8.6% 1.0 Reference	28.2% 4.18 (1.76, 9.93) **	
Absence	91.4% 1.0 Reference	71.8% 1.0 Reference	
Age, Mean, SD	68.55 (6.98) 1.0 Reference	68.55 (7.29) 1.00 (0.97, 1.03)	X^2^(1) = 0.00
Survivorship time (months) from diagnosis, Mean (SD)	71.17 (60.42) 1.0 Reference	54.66 (53.55) 0.99 (0.99, 1.00) *	X^2^(1) = 7.12 *
Household Income			X^2^(1) = 4.59_
*<CAD 50,000*	19.9% 1.0 Reference	30.7% 1.89 (1.01, 3.54) *	
*CAD 50,000–100,000*	47.0% 1.0 Reference	42.1% 1.09 (0.63, 1.87)	
*>CAD 100,000*	33.1% 1.0 Reference	27.2% 1.0 Reference	
	Good spiritual well-being (*n* = 130) 1.0 Reference	Poor spiritual well-being (*n* = 229) OR (95% CI)	Wald X^2^
Urinary function ^1^ (severity of lower urinary tract symptoms), UCLA ^2^, Mean (SD)	77.55 (19.79) 1.0 Reference	76.85 (22.11) 1.00 (0.99, 1.01)	X^2^(1) = 0.09
Bowel function ^1^ (severity) of bowel symptoms, UCLA ^2^, Mean (SD)	85.94 (16.34) 1.0 Reference	82.25 0.99 (0.97, 1.00)	X^2^(1) = 3.29
Sexual function ^1^ (severity of disfunction), UCLA ^2^, Mean (SD)	29.86 (29.25) 1.0 Reference	24.35 (25.57) 0.99 (0.98, 1.00)	X^2^(1) = 2.71
Physical health ^1^, SF-12 ^3^, Mean (SD)	48.97 (4.69) 1.0 Reference	47.35 (5.57) 0.94 0.90, 0.98) **	X^2^(1) = 7.48 **
Mental health ^1^, SF-12 ^3^, %	47.74 (3.92) 1.0 Reference	44.40 (5.65) 0.85 0.80, 0.90) ***	X^2^(1) = 28.33 ***
Treatment regret			X^2^(1) = 5.24 *
Presence	9.1% 1.0 Reference	23.2% 3.02 (1.17, 7.79) *	
Absence	90.9% 1.0 Reference	76.8% 1.0 Reference	
Age, Mean, SD	70.13 (6.62) 1.0 Reference	67.64 (7.26) 0.95 (0.91, 0.98) **	X^2^(1) = 8.82 **
Survivorship time (months) from diagnosis, Mean (SD)	76.36 (65.40) 1.0 Reference	55.76 (51.74) 0.99 (0.98, 0.99) **	X^2^(1) = 8.98 **
Household Income			X^2^(2) = 3.23
*<CAD 50,000*	22.1% 1.0 Reference	26.9% 0.978 (0.50, 1.91)	
*CAD 50,000–100,000*	51.9% 1.0 Reference	40.9% 0.63 (0.36, 1.12)	
*>CAD 100,000*	26.0% 1.0 Reference	32.2% 1.0 Reference	

* *p* < 0.05, ** *p* < 0.01; *** *p* < 0.001. ^1^ Lower score indicates worse function or health related quality of life. ^2^ University of California Los Angeles Prostate Cancer Index (UCLA PCI). ^3^ 12 Item Short form Survey (SF-12).

**Table 2:** Multivariate logistic regression assessing the relationship between social/family, emotional, functional and spiritual well-being outcomes and uro-oncological function, physical and mental health indicators, and demographic variables in a sample of men with a history of prostate cancer diagnosis from the baseline cycle of a Quality-of-Life Maritimes Survey administered between 2017 and 2020, *n* = 367.

	**Poor Social/Family Well-Being vs. Good Social/Family Well-Being (Reference)** **OR (95% CI)**	**Wald X^2^**
		X^2^(10) = 35.77
Urinary function ^1^ (severity of lower urinary tract symptoms), UCLA ^2^	1.02 (1.00, 1.039)	X^2^(1) = 3.82
Bowel function ^1^ (severity) of bowel symptoms, UCLA ^2^	0.98 (0.95, 1.02)	X^2^(1) = 0.86
Sexual function ^1^ (severity of disfunction), UCLA ^2^, Mean (SD)	0.99 (0.97, 1.00)	X^2^(1) = 1.88
Physical health ^1^, SF-12 ^3^	0.93 (0.85, 1.02)	X^2^(1) = 2.30
Mental health ^1^, SF-12 ^3^	0.90 (0.82, 0.99) *	X^2^(1) = 4.40 *
Treatment regret		X^2^(1) = 4.87 *
Presence	3.62 (1.16, 11.36) *	
Absence	1.0 Reference	
Age	1.02 (0.95, 1.08)	X^2^(1) = 0.28_
Survivorship time (months) from diagnosis	1.00 (0.99, 1.00)	X^2^(1) = 0.87
Household Income		X^2^(2) = 5.02
*<CAD 50,000*	3.77 (1.12, 12.65) *	
*CAD 50,000–100,000*	1.35 (0.50, 3.62)	
*>CAD 100,000*	1.0 Reference	
	**Poor Emotional Well-Being vs. Good Emotional Well-Being (Reference)** **OR (95% CI)**	**Wald X^2^**
		X^2^(10) = 42.95
Urinary function ^1^ (severity of lower urinary tract symptoms), UCLA ^2^	0.99 (0.97, 1.01)	X^2^(1) = 0.69
Bowel function ^1^ (severity) of bowel symptoms, UCLA ^2^	0.97 (0.95, 1.02)	X^2^(1) = 0.74
Sexual function ^1^ (severity of disfunction), UCLA ^2^	1.00 (0.98, 1.02)	X^2^(1) = 0.13
Physical health ^1^, SF-12 ^3^	0.91 (0.82, 1.01)	X^2^(1) = 3.21
Mental health ^1^, SF-12 ^3^	0.80 (0.71, 0.90)	X^2^(1) = 14.72 ***
Treatment regret		X^2^(1) = 8.45 **
Presence	5.58 (1.75, 17.75) **	
Absence	1.0 Reference	__
Age	1.00 (0.92, 1.09)	X^2^(1) = 0.01_
Survivorship time (months) from diagnosis	0.99 (0.98, 1.00)	X^2^(1) = 2.18
Household Income		X^2^(2) = 0.56
*<CAD 50,000*	1.12 (0.27, 4.73)	
*CAD 50,000–100,000*	0.72 (0.17, 2.94)	
*>CAD 100,000*	1.0 Reference	
	**Poor Functional Well-Being vs. Good Functional Well-Being (Reference)** **OR (95% CI)**	**Wald X^2^**
		X^2^(10) = 63.23
Urinary function ^1^ (severity of lower urinary tract symptoms), UCLA ^2^	0.99 (0.97, 1.01)	X^2^(1) = 0.36
Bowel function ^1^ (severity) of bowel symptoms, UCLA ^2^	0.99 (0.96, 1.03)	X^2^(1) = 0.13
Sexual function ^1^ (severity of disfunction), UCLA ^2^	0.98 (0.96, 0.99) *	X^2^(1) = 6.28 *
Physical health ^1^, SF-12 ^3^	0.82 (0.72, 0.92) **	X^2^(1) = 10.66 **
Mental health ^1^, SF-12 ^3^	0.77 (0.67, 0.87) ***	X^2^(1) = 15.84 ***
Treatment regret		X^2^(1) = 6.28 *
Presence	4.63 (1.40, 15.36) *	
Absence	1.0 Reference	
Age	1.04 (0.96, 1.11)	X^2^(1) = 0.96
Survivorship time (months) from diagnosis	0.99 (0.98, 0.99) *	X^2^(1) = 6.15 *
Household Income		X^2^(2) = 0.37
*<CAD 50,000*	0.72 (0.19, 2.72)	
*CAD 50,000–100,000*	0.71 (0.22, 2.26)	
*>CAD 100,000*	1.0 Reference	
	**Poor Spiritual Well-Being vs. Good Spiritual Well-Being (Reference)** **OR (95% CI)**	**Wald X^2^**
		X^2^(10) = 35.39
Urinary function ^1^ (severity of lower urinary tract symptoms), UCLA ^2^	1.00 (0.98, 1.02)	X^2^(1) = 0.22
Bowel function ^1^ (severity) of bowel symptoms, UCLA ^2^	1.01 (0.98, 1.04)	X^2^(1) = 0.44
Sexual function ^1^ (severity of disfunction), UCLA ^2^	1.00 (0.98, 1.01)	X^2^(1) = 0.14
Physical health ^1^, SF-12 ^3^	0.85 (0.76, 0.94) **	X^2^(1) = 9.18 **
Mental health ^1^, SF-12 ^3^	0.83 (0.73, 0.93) **	X^2^(1) = 9.67 **
Treatment regret		X^2^(1) = 1.53
Presence	2.09 (0.65, 6.71)	
Absence	1.0 Reference	
Age	0.97 (0.91, 1.03)	X^2^(1) = 0.90
Survivorship time (months) from diagnosis	0.99 (0.98, 0.99) *	X^2^(1) = 5.56 *
Household Income		X^2^(2) = 7.17 *
*<CAD 50,000*	0.18 (0.50, 0.67) *	
*CAD 50,000–100,000*	0.30 (0.10, 0.88) *	
*>CAD 100,000*	1.0 Reference	

* *p* < 0.05, ** *p* < 0.01; *** *p* < 0.001. ^1^ Lower score indicates worse function or health related quality of life. ^2^ University of California Los Angeles Prostate Cancer Index (UCLA PCI). ^3^ 12 Item Short form Survey (SF-12).

## Data Availability

Data from this study are available to researchers through a data access process.
